# Histological changes in odontogenic parakeratinized keratocysts treated with marsupialization followed by enucleation

**DOI:** 10.4317/medoral.23898

**Published:** 2020-10-09

**Authors:** Ugo Consolo, Giacomo Setti, Sara Tognacci, Chiara Cavatorta, Diana Cassi, Pierantonio Bellini

**Affiliations:** 1 Oral Medicine and Oral Surgery Unit. Department of Surgical, Medical, Dental and Morphological Sciences with Interest transplant, Oncological and Regenerative Medicine. Modena, Italy; 2 Pathologist. Anatomic Pathology, “M. Bufalini Hospital”, Cesena, Italy

## Abstract

**Background:**

The purpose of this study was to evaluate whether marsupialization treatment induces changes in the histology of odontogenic keratocyst epithelium and to compare our experience with the literature.

**Material and Methods:**

A retrospective revision of histological samples was performed. 5 patients with odontogenic keratocyst treated with marsupialization follow by enucleation were selected. Histologic evaluation analyzed the changes in the keratocyst epithelium after marsupialization in terms of type of keratinization, thickness of the epithelium and connective tissue, the presence of acanthosis, the presence and grade of fibrosis, the type and grade of inflammation and the presence and number of mitotic figures and daughter cysts.

**Results:**

In our case series, a variation of para-keratinized into ortho-keratinized keratocyst was found in one case, and no significant increases were observed in the epithelium and capsule thickness, or even in the level of inflammation. However, we observed an increase in fibrosis and qualitative changes in inflammation type.

**Conclusions:**

Minor and major histological changes were associated with reduction in cyst volume, which resulted in a simpler and less invasive cystic enucleation after marsupialization.

** Key words:**Keratocyst, marsupialization, enucleation, histology, histological changes.

## Introduction

Odontogenic keratocysts (OKCs) are locally aggressive lesions with high recurrence rate after surgical excision. OKC has been classified as an odontogenic cyst until 2005. Between 2005 and 2017 such lesion was classified as odontogenic keratocystic tumor (OKT) between tumors containing odontogenic epithelium with mature and fibrous stroma without odontogenic ectomesenchyme (World Health Organization (WHO) - Histological Classification for Odontogenic Tumors) (1). The 2017 revision of the WHO classification reclassified the OKC as a cystic lesion and renamed it “odontogenic keratocyst” (2).

A common radiographic feature of OKC is the unilocular, well-defined radiolucent aspect. In some cases, a multilocular patters is displayed (3).

OKC normally develops as a single lesion; when multiple lesions are concomitant, cysts are probably associated with a genetic mutation, eventually leading to nevoid basal cell carcinoma or Gorlin-Gotz syndrome (NBCCS) (4,5).

Compared to other odontogenic cysts, the histological appearance of OKC is rather peculiar. It consists of a uniformly thick cystic epithelial lining, composed of 6–10 cell layers with a well-polarized basal layer. Such epithelial layer tends to separate from the underlying connective tissue. The luminal surface exhibits feature of para-keratinization and is usually corrugated (6,7).

Several therapeutic options are available for OKC treatment, taking into account the recurrence rate and surgical morbidity. Radical therapy includes marginal resection or ‘en bloc’ resection; aggressive surgery yields the lowest rate of recurrence (8). Conservative solutions include marsupialization or decompression, both of which are followed by enucleation with or without adjunctive therapy, like Carnoy’s solution (7,9).

Marsupialization and decompression are the treatments of choice to reduce OKC volume prior to excision, as well as to minimize neurological and vascular complications, morbidity, and unaesthetic outcomes (10-12). Additionally, several authors have described cases involving marsupialization or decompression that resulted in histological changes in the cystic cell line, resulting in variations of an ortho-keratinized pattern or oral epithelium (12).

The purpose of the present investigation is to evaluate the histological variations in the epithelial lining of extensive mandibular OKC treated with marsupialization and enucleation.

## Material and Methods

- Cases presentation

Between 2013 and 2020, 14 cases of OKCs, 6 women and 5 men, were treated at the Dentistry and Oral-Maxillofacial Surgery Unit of the “Azienda Ospedaliera-Universitaria di Modena”, Modena, Italy. Four of the OKCs were associated to a diagnosis of NBCCS.

Treatment selection varied between 1) radical excision and 2) marsupialization plus excision according to patient age and compliance, lesion localization, extension, proximity to or involvement of peculiar anatomical structures (i.g. nervous, vascular), risk of recurrences, post-surgical morbidity and risk of fracture. Eight patients received marsupialization followed by excision (3 NBCCS and 5 non-NBCCS); 1 OKC recurrence was reported during follow-up (12,5%). Six OKCs were radically excised (1 NBCCS and 5 non-NBCCS) and 2 had recurrence during follow-up (33.3%).

The present analysis if focused on 5 mandibular OKC cases (3 females and 2 males), treated through marsupialization followed by enucleation, which were selected for histological retrospective analysis. Patients were aged between 27 and 62 years (mean 39.4 years). One patient was affected by NBCCS, the others having no comorbidities.

Case selection criteria were history of extensive osteolytic lesions of the mandibular angle and/or ramus, strict contact with inferior alveolar nerve (IAN), with partial or complete radiographic resorption of the bony canal; regular attendance to control appointments and complete radiographic history. All surgical interventions were performed by the same oral-maxillofacial surgeon. If present, associated impacted tooth were removed. Time between marsupialization and enucleation ranged from 7 to 34 months (Table 1).

- Surgical technique

Marsupialization was adequately planned after preoperative evaluation of orthopantomography and computer tomography (CT) scans. The purpose was to reduce lesion luminal pressure by opening a full thickness window, followed by suturing the cystic membrane to the surrounding oral mucosa.

Particularly, the endoluminal pressure reduction is necessary for volume shrinkage and separation from neurological structures.

Depending on the lesion morphology, the access window was performed on the mandible alveolar ridge (2 cases) or on the buccal side (3 cases). The removed cystic membrane was collected for histological examination.

The lesion lumen was washed repeatedly with hydrogen peroxide (3%) and chlorhexidine (0.2%); the cystic membrane was then sutured to the oral mucosa with a Vicryl 4.0 (Ethicon Inc.). The cystic cavity was filled with a space maintainer (gentamycin-soaked iodoform gauze (Mylan Generics, Italy)).

**Figure 1:**
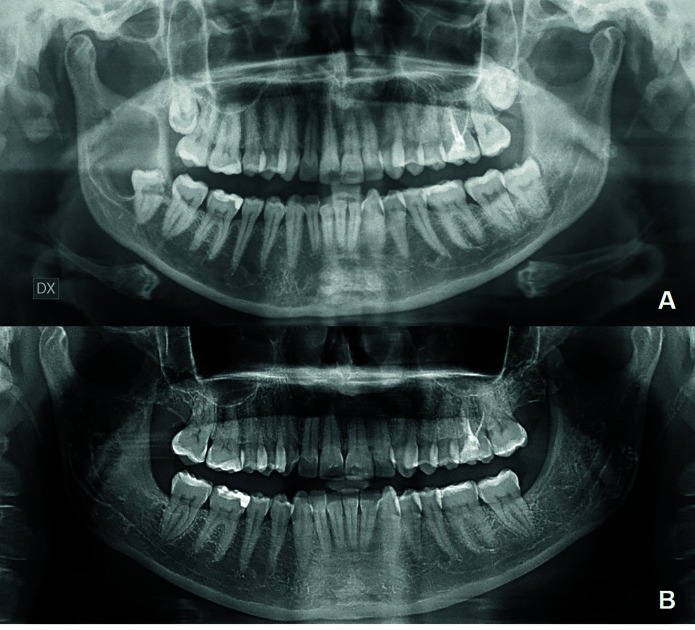
A) Radiographic examination at diagnosis. The lesion includes part of the unerupted 3rd molar and involves IAN with resorption and dislocation; B) Follow-up after 6 months from radical excision.

At weekly follow-ups, washes with hydrogen-peroxide and chlorhexidine were performed, followed by gauze substitution. Radiographic follow-up was carried out every 3 months, in order to schedule the cyst removal if mineralization of the cystic walls and thickening of the inferior alveolar nerve (IAN) canal were achieved. Following the second surgery, the excised cystic membrane was sent for histological examination. Patients were followed through radiographic and clinical examinations carried out once a year for the first 5 years and then every 2–3 years, according to the literature (Fig. 1) (13).

- Histological examination

All histological samples were included in paraffin and stained with hematoxylin and eosin (H&E). Specimens from the two surgical phases of each patient (marsupialization and excision) were retrieved and multiple slides were set up. Histological examinations were performed by a pathologist unaware of any previous diagnosis or treatment.

**Table 1:**
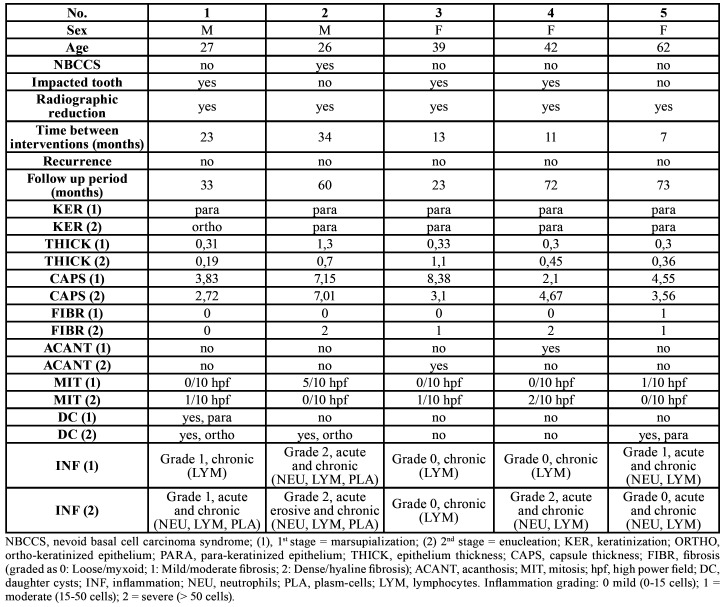
Patient data, clinical and histological features of treated OKCs. Descriptive histology is referred to: epithelium and capsule, fibrosis, acanthosis, mitotic figures, daughter cysts and grade of inflammation.

The 2017 WHO revision of the Diagnostic Criteria for Histological Characterization of OKCs was adopted (2). Specifically, examinations considered each the type of keratinization, thickness of the epithelium and connective tissue, the presence of acanthosis, the presence and grade of fibrosis, the type and grade of inflammation and the presence and number of mitotic Figures and daughter cysts.

Cell count and measures were performed on random selected high-power fields (HPF, 40x) on H&E slides.

**Figure 2:**
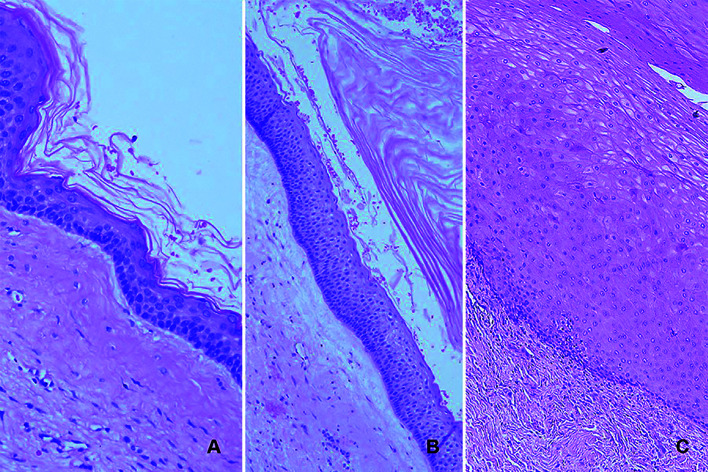
A) Hematoxylin and eosin (H&E), 20x, uniform thin para-keratinized epithelium loosely attached to the capsule (before marsupialization), grade 0; B) H&E, 10x, slide showing ortho-keratinized epithelium and mild fibrosis (after marsupialization), grade 1; C) H&E, 20x, acanthotic para-keratinized epithelium tightly attached to a dense fibrous capsule (after marsupialization), grade 2.

Fibrosis was graded according to thickness as loose/myxoid [0], mild/moderate fibrosis [1], dense/hyaline fibrosis [2] in accordance with the study of Awni et Conn (Fig. 2) (14).

Mitosis were also evaluated, including mitotic count per 10 HPF.

**Figure 3:**
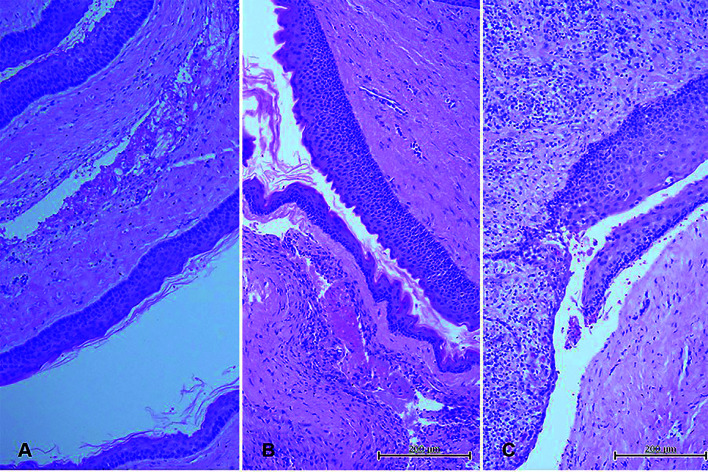
A) Hematoxylin and eosin (H&E), 10x mild inflammation (grade 1), with few cells present and scattered infiltration in the connective tissue; B) H&E, 10x, moderate inflammation, (grade 2), with inflammatory cells covering half of the field; C) H&E, 10x, severe inflammation (grade 3), inflammatory cells are dispersed in the entire field; particularly, giant cells are visible.

Depending on the type of infiltrating cells, inflammation was classified as acute when it was supported by polynucleated cells like neutrophils, and as chronic when mononucleated cells were present, such as plasma cells or lymphocytes. Inflammation was classified in according to Awni et Conn as mild [0] when 0 to 15 cells were present (scattered infiltration in the connective tissue), moderate [2] when inflammatory cells covered half of the field (15-50 cells) and severe [2] when inflammatory cells were dispersed in the entire field (>50 cells) (Fig. 3) (14).

## Results

All OKC samples exhibited para-keratinized epithelium at the time of marsupialization. At enucleation, one sample exhibited epithelial modification with the expression of an ortho-keratinized pattern.

Epithelium and fibrous capsule thicknesses changed without uniformity; the excision of two marsupialized cysts revealed overall epithelial thinning while three were thickened. Capsule thickness increased in one case, but capsules reduced in thickness in the four other samples (Table 1).

While fibrosis was scored as loose (grade 0) in 4 samples out 5, only one was scored as mild (grade 1). Fibrosis grading showed an irregular increase during the second histological evaluation; two cases exhibited a dense/hyaline fibrosis pattern, with an overall increase of two grades. One case showed an increase of one grade, with fibrosis change from loose/myxoid to mild/moderate. Conversely, two cases did not show any differences between surgical stages.

Acanthosis was present in one case before marsupialization and in one different case at cyst enucleation. Mitotic Figures were recognizable in two cases after the first surgery; the same lesions exhibited no mitotic activity at histopathological evaluation after enucleation slides. Conversely, three cases were negative for mitotic Figures but exhibited mitotic activity after enucleation (Table 1).

Daughter cysts with para-keratinized characteristics were observed in one case after marsupialization; after excision, daughter cysts with ortho-keratinized pattern could be observed. Furthermore, two other samples collected at the time of excision had daughter cysts, of which one manifested ortho-keratinized features and one para-keratinized ones. No daughter cysts were observed in the other cases.

At both marsupialization and excision, all cases exhibited an inflammatory infiltrate involving different type of cells; most noticeable was the presence of lymphocytes, which can be associated with other chronic inflammation cells like plasma cells or with acute inflammation cells like neutrophils. At the time of marsupialization, two samples exhibited acute inflammation, while the other samples had a mixture of acute and chronic inflammatory cells. In contrast, chronic inflammatory cells were found in just one sample after excision, while the other four samples had chronic and acute inflammation features. Additionally, comparison of inflammation grades at the time of the first and second surgeries revealed that three cases were unaltered, one case had an increased number of inflammatory cells, and in one case inflammation diminished (Table 1). Follow-up periods ranged from 23 to 73 months (mean 52.2 months), during which no recurrences occurred.

## Discussion

The treatment of choice for OKCs is still widely debated. Commonly, the therapeutic decision mainly takes into account the probability of recurrence and post-surgical morbidity (7). However, patient age, size and location of the cyst, soft tissue involvement, history of previous treatment as well as pattern of keratinization (when available on preoperative specimens) have been proposed as important parameters for choosing among treatment possibilities (15).

Marsupialization is based on two surgical stages, high patient cooperation, and a strict follow-up schedule between the two surgeries. Nevertheless, many clinicians choose this technique to reduce cyst volume before enucleation, and this approach can preserve perilesional teeth and bone, decrease the risk of inferior alveolar nerve injuries, and reduce morbidity and the risk of aesthetical and functional damage(16-21). Additionally, the recurrence rate of marsupialization is not higher than primary radical excision (21).

According to Woolgar *et al*., recurrences can result from three situations: incomplete removal of cystic membrane; growth of a new cyst from daughter or satellite cysts or odontogenic epithelial cysts; development of an unrelated OKC in an adjacent region (4).

Marker *et al*. evaluated the histological features of 23 OKCs treated with decompression followed by enucleation and reported that decompression induces shrinkage of the cyst volume, new bone formation, and thickening of the cyst wall; these modifications facilitate enucleation and lower the risk of incomplete cyst wall removal (12). Telles *et al*. described similar findings on six OKCs after morphometric analysis of the epithelial lining and fibrous capsule. According to their study, thickness of the epithelial lining and of the fibrous capsule increased significantly after marsupialization, leading to easier surgical enucleation and a reduced recurrence rate (22). Nakamura *et al*. also reported that marsupialization reduced the cyst size and the extent of surgery but did not modify the recurrence rate of OKCs (23).

Awni and Conn described the behavior of 17 OKCs treated with decompression or marsupialization or a combination of decompression/marsupialization and surgical excision. All the lesions were biopsied twice, at different stages to evaluate histological variations. Regarding to fibrosis, the authors described a reduction in separation of the epithelium from the underlying fibrous capsule after decompression, suggesting that these features are considered to facilitate surgical removal, lowering recurrence rate by reducing the chances of leaving OKCs epithelium (14).

With the limitation of a small sample size, our case series showed similar results considering the marsupialization treatment, with an irregular increase; epithelial thickening was appreciable in 3 cases only and capsule thickening was described in 1 case out of 5. Fibrosis varied from a loose/myxoid pattern (grade 0) in 3 patients out of 5: two samples exhibited a dense/hyaline fibrosis (grade 2) and one mild/moderate (grade 1).

The second condition influencing recurrence rate is the presence of daughter cysts, which has been described by several authors. Marker *et al*. reported daughter cysts as sporadic findings, while Nakamura *et al*. examined 28 OKCs treated through a two-stage protocol and found micro-cysts or epithelial islands in six lesions before marsupialization and in 10 cysts after excision without specifying the keratotic pattern (12,23). In addition, Awni and Conn reported about an increase of daughter cyst number after decompression (14).

In the present case series, one specimen had a para-keratinized daughter cyst at the time of marsupialization, and daughter cysts with a mutation in the ortho-keratinized pattern were identified in specimens following excision. The other four cases had no daughter cysts at the time of marsupialization, but at the time of excision one had daughter cysts with a para-keratinized pattern, one had a daughter cyst with an ortho-keratotic pattern, and two had no daughter cysts.

A commonly reported histological change after marsupialization is the modification of OKC parakeratotic epithelium into oral-lining epithelium or ortho-keratinized epithelium. Awni and Conn, reported that seven of the 17 cases (41%) of the series, showed loss of para-keratinization, with two cases showing complete change to a nonkeratinized lining after decompression; however, results were not statistically significant and not related to duration of treatment (14). Nakamura *et al*. described 28 OKCs, of which 23 were identified as parakeratinized, one as ortho-keratinized, three a mixture of the two patterns, and one as unclassifiable. At the time of excision, they reported that 10 marsupialized OKCs had lost the histological features of the original lesion, exhibiting a hyperplastic, stratified, non-keratinized squamous epithelium and thick connective tissue. They also reported that two lesions had a combination of parakeratotic and orthokeratinized epithelium and nine were unchanged with a parakeratotic pattern (23).

Pogrel examined 10 OKCs for histological and immune-histochemical diagnosis and obtained similar findings: excision following marsupialization returned specimens appearing as oral epithelium, with no signs of cystic residues, daughter cysts, or basal layer budding (11).

August *et al*. conducted similar examinations on 14 OKCs, found that the histological features of OKCs disappeared in nine cases (24). Similar to other reports, authors suggested that the change of the para-keratinized epithelial lining into a less aggressive pattern is due to the presence of inflammation that occurs after marsupialization (22,23).

Our case series all exhibited para-keratinized epithelium during the first surgery, and a variation into ortho-keratinized epithelium was observed in one patient only. Rate of OKCs lining variation from para-keratinized into ortho-keratinized (20%) was lower in comparison with literature (14,25,26) but it is worth of mention a similar percentage of OKCs lining change (2 of 6, 33%) in the case series described by Telles *et al*. (22).

Moreover, inflammation scores were increased in one case: acute phase cells were the most abundant after marsupialization, indicating that a change in the inflammatory infiltrate occurred qualitatively and not quantitatively.

Additionally, de Paula *et al*. noticed increased proliferative activity in OKCs with abundant inflammatory infiltration. In our series, mitotic Figures were present in three samples after enucleation, but not at the time of the first surgery. Conversely, one case exhibited increases in both quantitative inflammation and mitotic Figure count (25). However, using the Ki-67 stain, Nakamura *et al*. demonstrated that proliferative activity was lower after excision than after marsupialization. They also suggested that the proliferation index is not influenced by the presence of daughter cysts or epithelial islands (23).

Some authors have further evaluated immunohistochemical modifications such as cytokeratin 10, Bcl-2 gene expression, CD34 expression related subepithelial angiogenesis as predictor of recurrences or have performed analyses with anti-Ki-67 antibody, but these were beyond the scope of our investigation (14,21,25,26).

With regard to therapeutic strategies and recurrence rates, scientific debate for a gold standard treatment option continues; the 2018 systematic review of de Castro *et al*. reported marsupialization followed by enucleation amongst the best conservative treatments for OKCs, due to the low recurrence rate (17.8%; 95% CI = 6.6 to 28.9%) (27). Our cases series exhibited no recurrence after a mean 52.2-month follow-up period.

De Castro *et al*. also suggested that time of marsupialization must be at least 9 months in order to obtain histological changes and relate clinical outcomes (27). Awni and Conn identified a significant increase in p53 expression, inflammation changes and daughter cysts development suggesting that OKCs becomes more hostile in nature with prolonged marsupialization time; moreover, epithelial atrophy was described in prolonged decompressions (14). Nevertheless, it is described that occasionally short timeframes are sufficient in order to appreciate major histological changes in OKCs (22).

Several authors reported of variable period between surgical stages (1.5 to 48 months), influenced by lesion dimension, patients age and compliance (11,14,22-24).

This retrospective analysis reported that epithelial and capsule thickness augmentation, fibrosis grade increase and inflammation grade improvement were appreciable in one case with a 11 months interval between surgeries. Longer period (23 and 34 months) were related to thinning of lining epithelium and capsule; likewise, the daughter cysts number had increased, while inflammation scores did not.

Despite of the small sample size, it is possible to speculate that longer treatment timeframes are necessary for extensive lesions in order to obtain considerable bone healing. On the other hand, subjective inflammatory response and prolonged marsupialization period could affect epithelium and capsule thickness.

All the above-mentioned histological findings could have been biased by the incidental and random selection of a part of the lesion. After radical excision the lesions were overall evaluated, allowing the pathologist to describe all detecTable characteristics. Conversely, the first surgical stage influences the comparison, providing a precise but unavoidable focal description of disease features.

## Conclusions

Main limitations of this study were the retrospective observational nature, the absence of a control group and the limited sample size. Minor and major histological changes were associated with reduction in cyst volume, which resulted in a simpler and less invasive cystic enucleation after marsupialization. However, more research is needed with a larger study population and a follow-up period of longer than 5 years to confirm these results.
